# Phagocytosis of *Aspergillus fumigatus *conidia by primary nasal epithelial cells *in vitro*

**DOI:** 10.1186/1471-2180-8-97

**Published:** 2008-06-18

**Authors:** Françoise Botterel, Karine Gross, Oumaïma Ibrahim-Granet, Khaled Khoufache, Virginie Escabasse, André Coste, Catherine Cordonnier, Estelle Escudier, Stéphane Bretagne

**Affiliations:** 1 Université Paris 12, Créteil, France; 2 Unité des Aspergillus, Institut Pasteur, 75724, Paris, France; 3 Inserm U 492, and Université Paris 12, Créteil, France; 4 Service d'ORL et de Chirurgie Cervico-Faciale, Hôpital Intercommunal de Créteil, France; 5 Service d'Hématologie Clinique, Hôpital Henri Mondor (AP-HP), Créteil, France; 6 Département de Génétique, Cytogénétique et Embryologie, Groupe Hospitalier Pitié-Salpêtrière (AP-HP), Paris, France; 7 Laboratoire de Parasitologie-Mycologie, Hôpital Henri Mondor, 51 avenue du Général De Lattre de Tassigny, 94010 Créteil, France

## Abstract

**Background:**

Invasive aspergillosis, which is mainly caused by the fungus *Aspergillus fumigatus*, is an increasing problem in immunocompromised patients. Infection occurs by inhalation of airborne conidia, which are first encountered by airway epithelial cells. Internalization of these conidia into the epithelial cells could serve as a portal of entry for this pathogenic fungus.

**Results:**

We used an *in vitro *model of primary cultures of human nasal epithelial cells (HNEC) at an air-liquid interface. *A. fumigatus *conidia were compared to *Penicillium chrysogenum *conidia, a mould that is rarely responsible for invasive disease. Confocal microscopy, transmission electron microscopy, and anti-LAMP1 antibody labeling studies showed that conidia of both species were phagocytosed and trafficked into a late endosomal-lysosomal compartment as early as 4 h post-infection. In double immunolabeling experiments, the mean percentage of *A. fumigatus *conidia undergoing phagocytosis 4 h post-infection was 21.8 ± 4.5%. Using combined staining with a fluorescence brightener and propidium iodide, the mean rate of phagocytosis was 18.7 ± 9.3% and the killing rate 16.7 ± 7.5% for *A. fumigatus *after 8 h. The phagocytosis rate did not differ between the two fungal species for a given primary culture. No germination of the conidia was observed until 20 h of observation.

**Conclusion:**

HNEC can phagocytose fungal conidia but killing of phagocytosed conidia is low, although the spores do not germinate. This phagocytosis does not seem to be specific to *A. fumigatus*. Other immune cells or mechanisms are required to kill *A. fumigatus *conidia and to avoid further invasion.

## Background

Invasive aspergillosis (IA) has a high case-fatality rate [1], and the prevalence of this fungal infection has increased in parallel with the number of at risk patients [2,3]. IA is an opportunistic infection resulting from inhalation of conidia released by *Aspergillus fumigatus*, the main species responsible for this disease [4]. Due to their small size (2–3 μm in diameter), these conidia can reach the alveoli where they are normally phagocytosed and killed by alveolar macrophages through the release of reactive oxidant intermediates [5]. Intracellular trafficking of *A. fumigatus *within alveolar macrophages has been described [6]. A defect in these alveolar macrophage functions could result in the germination of conidia and subsequent tissue invasion by fungal hyphae.

In contrast to alveolar macrophages, the role of airway epithelial cells in protection against IA has not been studied extensively, despite the fact that these are the first cells of the respiratory tract to encounter inhaled conidia. Moreover, the airways epithelium is frequently damaged by chemotherapy, radiation, viral or bacterial infections in immunocompromised patients at risk of IA [7,8]. This damage could trigger invasion from this site. Binding and internalization of *A. fumigatus *conidia by cells which are not professional phagocytes has already been demonstrated in A459 lung epithelial cells [9,10] and endothelial cells [11]. Rabbit tracheal epithelial cells have also been shown to internalize *A. fumigatus *conidia but this internalization has not been quantified [9,12].

In order to study the interaction between *A. fumigatus *conidia and the respiratory epithelium, an *in vitro *model of human nasal epithelial cells (HNEC) at an air-liquid interface has been developed [13-15]. The HNEC culture is a primary culture model in which the air-liquid interface results in airway cell differentiation [16]. After 1 week of culture, HNEC are organized into a pseudo-stratified epithelium with mucus and ciliated cells, and flow cytometry analysis of cytokeratin immunofluorescent labeling has demonstrated 99% positive cells, thus excluding the presence of other cell types [15]. The surface epithelium of the nose resembles that of the lower airways and HNEC are thus representative of the airways epithelium [16]. Moreover, HNEC are also representative of sinonasal epithelium and sinuses are the second anatomical site involved in IA [1,17,18].

We used this model to quantify internalization of *A. fumigatus *conidia by HNEC. As *A. fumigatus *is the main species responsible for IA [4], we wondered whether the rate of internalization of *A. fumigatus *conidia was different from other mould species and could explain, at least in part, the virulence of this opportunistic fungus. Virulence of *A. fumigatus *is achieved despite the fact that this fungus is usually outnumbered by other species in the environment [19]. Therefore, as a control we chose to use *Penicillium chrysogenum*, a fungus which is highly prevalent in the environment and which has a similar conidial size (3–4 μm in diameter) to *A. fumigatus*. *P. chrysogenum *is able to grow at 37°C [20] but is rarely involved in invasive disease [21].

## Results

To speed up contact between HNEC and fungal conidia, the HNEC were centrifuged and culture medium containing the conidia was immediately removed from the apical side to restore the air-liquid interface, because immersion rapidly leads to dedifferentiation of HNEC. This centrifugation had no effect on the electrophysiological parameters of the HNEC. Given the limited number of cells recovered from the nasal polyps, the number of available wells differed between primary cultures. Therefore, data could not be recorded for all observation times for every batch of HNEC.

**Figure 1:**
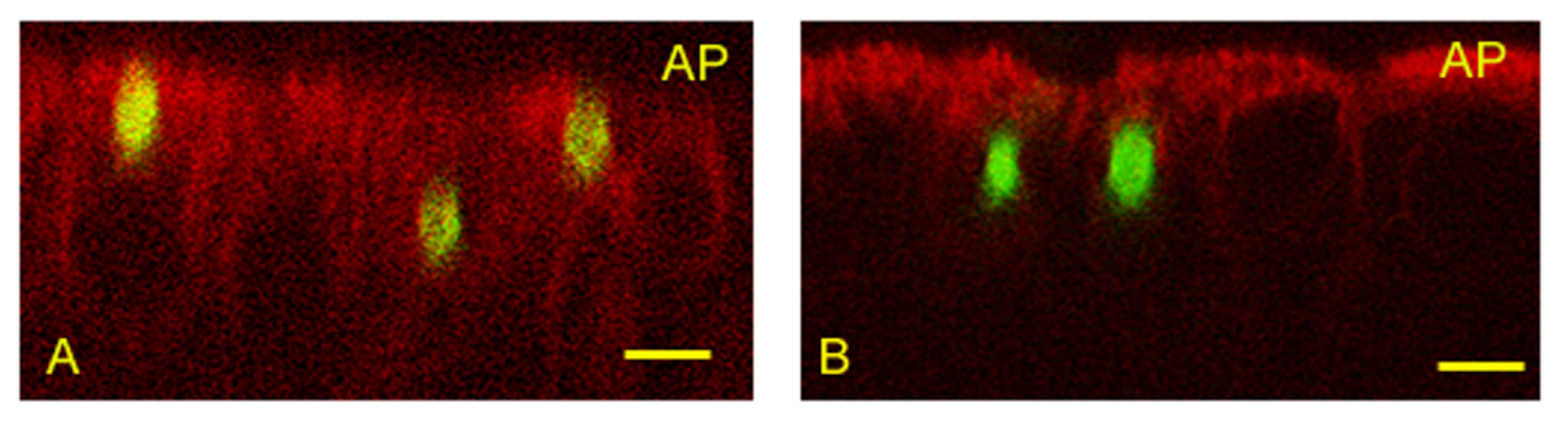
**Z view of HNEC showing internalization of *A. fumigatus *and *P. chrysogenum *conidia using confocal immunofluorescence microscopy.** Cells were incubated with FITC-labeled conidia for 8 h. The cells were then labeled with specific human cytokeratin antibody and conjugated secondary antibody. (A) Three *A. fumigatus *conidia in HNEC. (B) Two *P. chrysogenum *conidia in HNEC. AP: apical pole. Bar = 5 μm.

**Figure 2:**
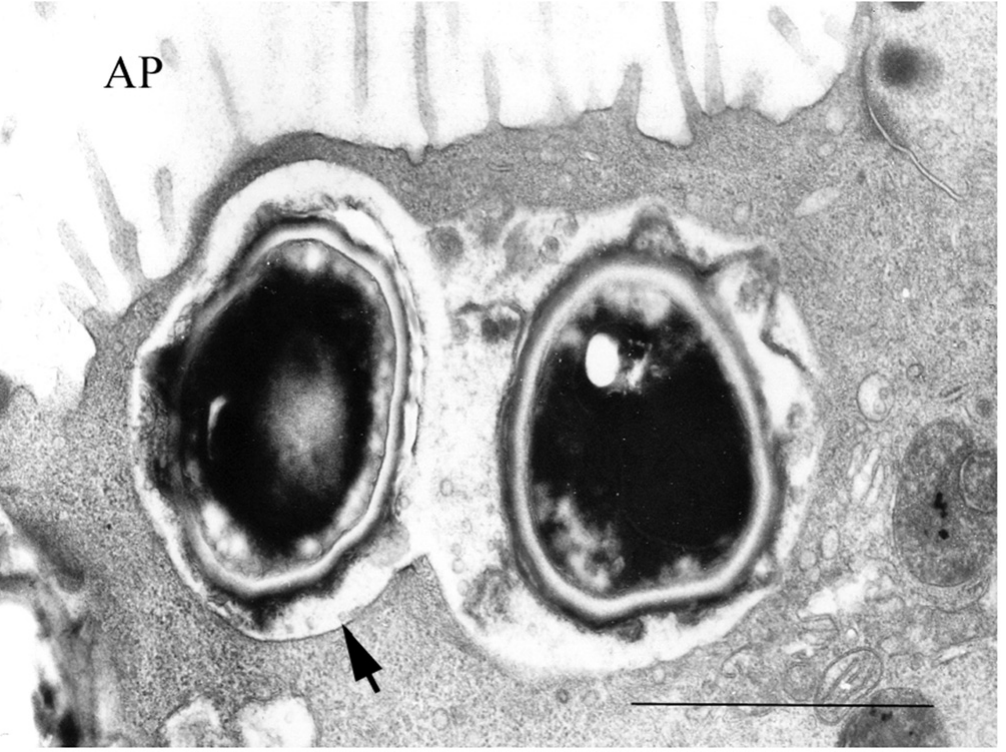
**Transmission electron microscopy of HNEC after 4 h incubation with *A. fumigatus *showing conidia internalized in a membrane-bound vacuole.** Note the double-layered cell wall (arrow) and the electron-dense pigmented outer layer of the conidium. AP: apical pole. Bar = 1 μm.

**Figure 3:**
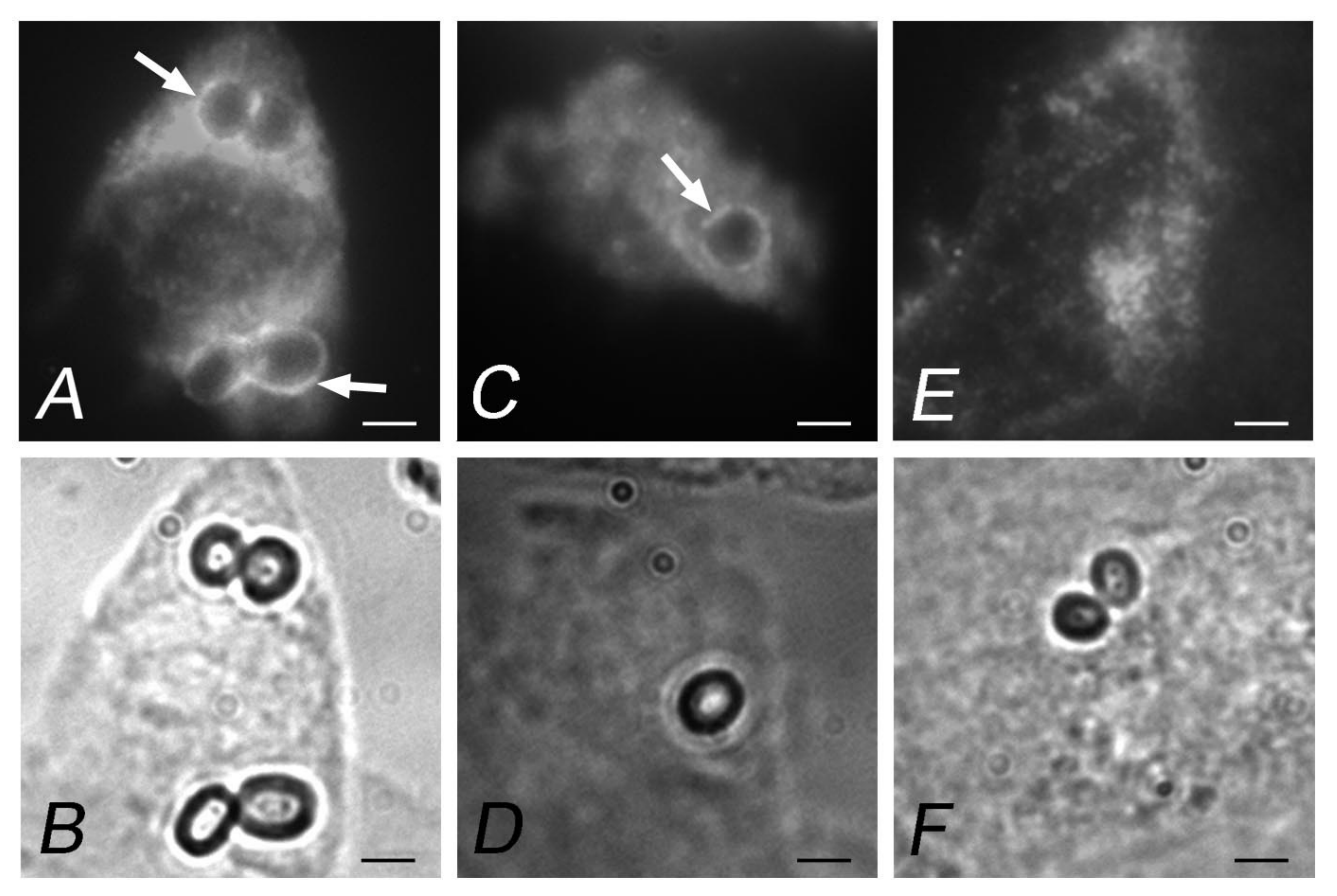
**Intracellular trafficking of *A. fumigatus *and *P. chrysogenum *conidia within HNEC phagosomes after 4 h incubation.** After fixation and permeabilization, cells were labeled with specific mouse monoclonal anti-LAMP1 antibody (mAb) and secondary antibody conjugated to Texas-red. The lower figures are the corresponding phase contrast images. (A and B) *A. fumigatus *conidia; (C and D) *P. chrysogenum *conidia; (E and F) control HNEC labeled with mouse IgG1 mAb. Arrows indicate conidia stained positive for LAMP1. Bar = 2 μm.

Preliminary studies, using confocal microscopy with Z view, suggested that *A. fumigatus *and *P. chrysogenum *conidia were internalized after 8 h of contact with HNEC (Figure 1). This was confirmed by transmission electron microscopy (TEM), which showed that *A. fumigatus *conidia were internalized into vacuoles (Figure 2). Staining of lysosomal LAMP1 membrane protein revealed the mature phagosomes of the conidia-containing vacuoles. Lysosomal LAMP1 membrane protein is acquired by late endosomes during their maturation to lysosomes [6]. A positive immunofluorescence signal was detected around ingested *A. fumigatus *and *P. chrysogenum *conidia as early as 4 h after infection (Figure 3). The mouse IgG1 isotype control antibody did not label any vacuole-like structures. These findings provide evidence of late endosomal-phagolysosomal trafficking of internalized *A. fumigatus *conidia.

**Figure 4:**
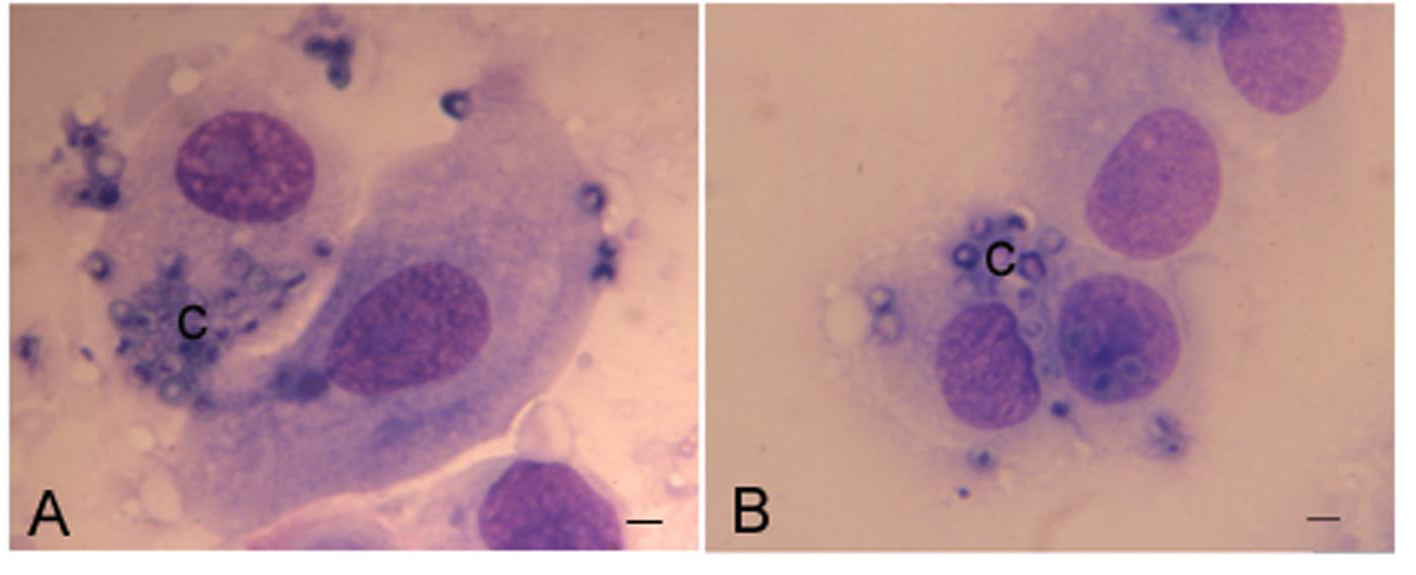
**HNEC stained with May-Grünwald Giemsa after 8 h incubation with *A. fumigatus *(A) and *P. chrysogenum *(B) conidia.** Cells were released and smears prepared before staining. Quantification of HNEC and conidia was performed to determine the number of HNEC-associated conidia and the average number of conidia associated with HNEC. (c) = conidia. Bar = 2 μm.

Confocal microscopy and TEM did not allow us to quantify the number of bound and phagocytosed conidia since HNEC do not form a monolayer and the cells are tightly bound together. As a consequence, the number of individual epithelial cells in contact with the conidia cannot be counted. Therefore, we dissociated the HNEC after contact with unlabeled conidia. Among 100 randomly selected HNEC, observed in two independent experiments, and 20% (± 4) and 18% (± 6) were associated with at least one conidium after 8 h interaction between HNEC and conidia, with between 1 and 16 conidia per cell (mean = 6.18) and 1 and 10 conidia per cell (mean = 5.74) for *A. fumigatus *and *P. chrysogenum*, respectively (Figure 4). The differences between the two fungi were not statistically significant.

**Figure 5:**
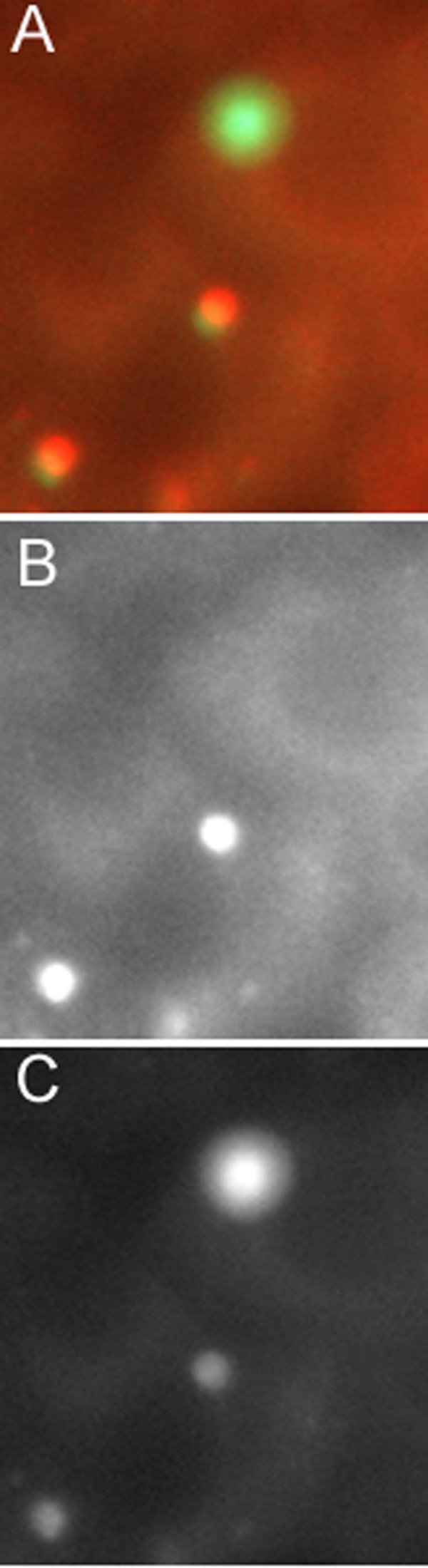
**Internalization of *A. fumigatus *conidia by HNEC.** HNEC were incubated with FITC-labeled *A. fumigatus *conidia for 4 h, washed to remove unbound conidia, and fixed. HNEC were incubated with a rabbit anti-conidia antiserum and then with Texas-red-conjugated anti-rabbit antibody to label non-phagocytosed conidia. (A) Overlay of green and red channels; (B) red channel (non-phagocytosed conidia); (C) green channel (total conidia). Bar = 2 μm.

However, these experiments did not distinguish between bound and phagocytosed conidia. To quantify phagocytosis of *A. fumigatus *by HNEC, two different experiments were performed. The first involved FITC-labeled conidia and anti-*A. fumigatus *conidia antiserum (Figure 5). The proportion of *A. fumigatus *conidia phagocytosed by HNEC was 21.8 ± 4.5% after 4 h of contact in three different primary cultures (Table 1). As no anti-*P. chrysogenum *conidia antiserum was available, this experiment was not performed with this fungus.

**Table 1:** Extent of internalization of *A. fumigatus *conidia by different HNEC cultures after 4 h contact.

HNEC culture	Bound conidia (%)	Internalized conidia (%)
C143 P	8.5	19.5
C149 P	7.1	27.0
C153 P	7.8	19.0
Mean	7.8 ± 0.7	21.8 ± 4.5

Internalization was revealed by double immunostaining. Percentages and means were calculated from the bound conidia figures for two wells of three different primary cultures.

The second experiment to quantify phagocytosed conidia used the fluorescent brightener, Blankophor-P-fluessig^® ^(4,4'-Bis [(4-anilino-6-substituted-1,3,5-trazine-2-yl)] amino stilbene-2,2'-disulfonic acid), which is a similar fluorescent diaminostilbene compound to Calcofluor white and Uvitex 2B [22]. Quantification of phagocytosis with seven different HNEC cultures is shown in Table 2. The proportion of conidia taken up varied between 9% and 33% for *A. fumigatus *(mean: 18.7 ± 9.3%) according to the primary culture used. A similar or non-significant increase in phagocytosis rate was observed with longer observation times up to 20 h (Table 2). After 24 h, observation was hampered by the growth of hyphae from non-internalized conidia. We investigated the internalization of *A. fumigatus *and *P. chrysogenum *conidia in parallel in two primary cultures (Table 2). No statistically significant difference (*p *> 0.5) was observed between the two species for a given culture, irrespective of the observation time.

**Table 2:** Extent of phagocytosis of *A. fumigatus *and *P. chrysogenum *conidia by HNEC revealed using the fluorescence brightener Blankophor to stain non-phagocytosed conidia.

			Contact time			
HNEC culture	Fungal species	Bound conidia (%)	4 h	8 h	12 h	20 h
C313P	*A. fumigatus*	12.2	18.4	33.4	Nd	Nd
C321P	*A. fumigatus*	14.4	8.3	12.5	nd	nd
C338P	*A. fumigatus*	12.0	12.8	28.6	nd	nd
C349P	*A. fumigatus*	9.9	nd	14.5	19.2	38.1
C350P	*A. fumigatus*	9.5	nd	20.9	16.1	27.6
C360P^a^	*A. fumigatus*	8.6	nd	11.5	9.9	20.9
C367P^a^	*A. fumigatus*	7.5	nd	9.2	9.7	20.3
Mean		10.6 ± 2.4	13.2 ± 5.1	18.7 ± 9.3	14.0 ± 4.7	26.7 ± 8.3
C360P^a^	*P. chrysogenum*	8.9	nd	9.0	11.9	17.2
C367P^a^	*P. chrysogenum*	6.4	nd	10.2	10.5	18.3
Mean		7.7		9.6	11.2	17.8

Percentages and standard deviations were calculated from the bound conidia figures for two wells at each time point with seven and two different primary cultures for *A. fumigatus *and *P. chrysogenum *respectively.

nd: not done

^a^*p *value > 0.05 with the non-parametric Kruskall Wallis test for the two species for the same primary culture.

During our experiments, we were unable to detect any germinated conidia inside HNEC until 20 h post-infection. In addition, we also failed to detect any modifications to the electrophysiological parameters of the HNEC. This indicates that HNEC inhibited germination of phagocytosed conidia and had a fungistatic or fungicidal effect. To study the rate of killing of phagocytosed conidia, we used a combination of propidium iodide (PI) and Blankophor staining. The results are summarized in Table 3. The killing rate for *A. fumigatus *conidia varied from 9–24% (mean: 16.7 ± 7.5%) after 8 h according to the HNEC culture. With longer incubation times the killing rate usually increased, without exceeding 35%. The killing rate of *P. chrysogenum *conidia was lower than with *A. fumigatus *conidia for a given HNEC (Table 3).

**Table 3:** Killing of phagocytosed conidia revealed by propidium iodide staining.

			Killing rate (%)		
HNEC culture	Fungal species	Bound conidia (%)	8 h	12 h	20 h
C349P	*A. fumigatus*	9.9	11.3	25.3	35.0
C350P	*A. fumigatus*	9.5	9.3	40.0	47.3
C360P^a^	*A. fumigatus*	8.6	24.3	29.2	30.5
C367P^a^	*A. fumigatus*	7.5	22.0	24.7	29.9
Mean		8.8 ± 1.1	16.7 ± 7.5	30.0 ± 7.1	35.7 ± 8.1
C360P^a^	*P. chrysogenum*	8.9	11.7	13.5	15.3
C367P^a^	*P. chrysogenum*	6.4	6.3	14.7	18.8
Mean		7.7	9.0	14.1	17.0

Percentages and standard deviations were calculated from the bound conidia figures for two wells at each time point with four and two different primary cultures for *A. fumigatus *and *P. chrysogenum *respectiveley.

^a^*p *value < 0.05 with the non-parametric Kruskall Wallis test for the two species for the same primary culture.

## Discussion

Using an original *in vitro *model of HNEC at an air-liquid interface, we showed that HNEC are able to phagocytose *A. fumigatus *conidia. Labeling of lysosomal LAMP1 membrane protein confirmed that internalized conidia were trafficked into a late endosomal-lysosomal compartment. Phagocytosis of *A. fumigatus *conidia by alveolar macrophages is close to 100% [6], but is much lower for non-professional phagocytes such as the A549 cell line and human umbilical vein endothelial cells for which phagocytosis rates of 30% and 50%, respectively, have been reported [10]. In the present study, the rate of conidia phagocytosed by HNEC was 21.8 ± 4.5% at 4 h post-infection using double immunolabeling. Using combined staining with a fluorescence brightener, the mean rate of conidia phagocytosed was 18.7 ± 9.3%. The differences observed between different primary HNEC cultures and these two different methods might be due to difficulties in standardizing the inoculum, contact between conidia and HNEC, the number of cells in each well, and the ratio between the different cell types. Indeed, in some instances high standard deviations were observed, calculated from the means of three wells. However, irrespective of the HNEC culture the phagocytosis rate increased slightly between 4 and 8 h, and remained stable until 20 h for a given culture. Another explanation for the differences observed between HNEC could be the genetic background of the patients, since these HNEC are primary cultures. Indeed, the phagocytosis rate may depend on some intrinsic cellular properties. This may explain the lack of reproducibility of the experiments. On the other hand, it may also explain why some patients develop invasive disease and others do not, as suggested by studies on innate immunity in IA [23].

Using a fluorescence brightener and PI uptake [24], we confirmed that > 75% and > 50% of the conidia were viable after 8 h and 20 h of contact, respectively. Although HNEC were unable to kill most phagocytosed conidia, germination of the conidia inside the HNEC was inhibited for at least 20 h after phagocytosis. In cell-free media, swelling and germination of *A. fumigatus *conidia occurs after as little as 6–8 h of culture at 35°C [25], as observed with the reference strain used (data not shown). This suggests that HNEC inhibited germination of phagocytosed conidia and had a fungistatic effect. At longer incubation times, observation was hampered by hyphae from non-phagocytosed conidia growing over the HNEC. Therefore, we cannot exclude the possibility that germination of phagocytosed conidia occurs after 24 h inside the cells or outside the cells after phagocytosis. Similar observations have been reported with A549 cells. Whereas none of the phagocytosed conidia germinated after 6 h [10], 34% germinate after 24 h [26]. Additional experiments are needed to clarify the fungistatic effect of HNEC lysosomes and the ability of fungal conidia to survive in phagolysosomes.

Comparison of our results with *A. fumigatus *conidia and those with *P. chrysogenum *suggest that phagocytosis of the two species does not differ significantly for a given primary culture. HNEC are known to internalize bacteria [27] and inert particles [28]. We have thus extended this observation to fungal conidia. We did not observe better adhesion of *A. fumigatus *conidia to HNEC compared to *P. chrysogenum *conidia. However, a slight difference was observed in the killing rates. Killing of *P. chrysogenum *conidia was lower for a given HNEC. This might be explained by the slower growth rate of *P. chrysogenum *at 37°C compared to *A. fumigatus *if the cells only have anti-conidial activity against germinating elements. Therefore, the higher prevalence of *A. fumigatus *as a cause of IA compared to other *Aspergillus *species is probably not explained by better adhesion of conidia to the cells or a higher rate of phagocytosis. Nevertheless, other factors such as complement [29] could affect the conidia *in vivo *and modify phagocytosis and killing rates differently according to the fungal species.

## Conclusion

Using for the first time HNEC to study interactions between conidia from different species and respiratory epithelial cells, our data provide evidence that respiratory epithelial cells can phagocytose *A. fumigatus *and *P. chrysogenum *conidia. The phagocytosis rate did not differ between *A. fumigatus *and *P. chrysogenum *for a given primary culture. Moreover, the cells do not rapidly kill these conidia and uptake of conidia into nasal epithelial cells in primary culture was not accompanied by apparent cell destruction. Thus, phagocytosed conidia may serve as a reservoir for dissemination throughout the host. The differences in uptake of *A. fumigatus *conidia observed between different primary cultures may arise from a difference in individual host cell response to conidial exposure.

## Methods

### Fungal strains and preparation of conidia

Conidia were obtained from *A. fumigatus *strain IP 2279.94 (Pasteur Institute), originally isolated from a patient with IA, and from *P. chrysogenum *strain IP 1652.86 (Pasteur Institute) isolated from the environment. Fungi were cultured on 2% malt agar slants (Bio-Rad, Marnes-la-Coquette, France). Conidia were harvested after 7 days at 30°C by rinsing the slants with phosphate-buffered saline (PBS) supplemented with 0.1% Tween 20. Conidia were obtained after filtration through a 40 μm pore-size cell strainer (Millipore, Molsheim, France) to remove mycelium and suspended in cell culture medium.

### HNEC primary cultures and infection with conidia

The HNEC culture was adapted from a culture model originally developed with human tracheo-bronchial cells [30]. Nasal polyps were harvested from patients with nasal polyposis undergoing ethmoidectomy at the Intercommunal Hospital of Créteil. Preparation of HNEC cultures was performed as described previously [13,14]. The transport medium, DMEM/F12 (Invitrogen, Cergy-Pontoise, France), consisted of a mixture of Dulbecco's modified Eagle's nutrient medium (Life Technologies, France) and Ham's-F12 nutrient medium (Life Technologies, France) supplemented with antibiotics (100 U/ml penicillin, 100 mg/ml streptomycin, 100 mg/ml gentamicin, 2.5 μg/ml amphotericin B). The polyps were immediately transported to the laboratory and stored at 4°C for 2 h. Each nasal polyp was rinsed in PBS-antibiotics with 5 nM dithiothreitol and placed overnight at 4°C in a PBS-antibiotics solution containing 0.1% pronase (Sigma, France). The sample then was rinsed in DMEM/F12 with 5% fetal calf serum (FCS). After centrifugation (1000 g, 7 min), the cell pellets were suspended in a 0.25% trypsin solution (Life Technologies, France) diluted in DMEM/F12 with 5% FCS for 3 min, centrifuged and then suspended in DMEM/F12-5% FCS. Finally, 10^6 ^cells were plated in 12-mm insert wells (Transwell, Costar, Dutscher, France) with micropore membranes coated with type IV collagen (Sigma, France) and incubated at 37°C in 5% CO_2_. After 24 h, the liquid medium was removed from the apical compartment to obtain an air-liquid interface. The basal compartment was filled with 1 ml of HNEC culture medium, consisting of DMEM/F12 with 2% ultroser G (Life Sciences, Cergy-Pontoise, France) with antibiotics (100 U/ml penicillin, 100 mg/ml streptomycin, 100 mg/ml gentamicin). The HNEC culture medium was changed daily and the electrophysiological properties of the HNEC were checked twice a week using a microvoltmeter (EVOM^® ^World Precision Instruments, Aston-Stevenage, UK). All experiments were performed on 14-day-old HNEC cultures in which cell differentiation was well established.

Each HNEC well was seeded with 10^7 ^conidia. To speed up sedimentation and to avoid prolonged immersion of the cells, the wells were centrifuged at 60 g for 5 min and washed three times to remove unbound conidia. For each experiment, the number of conidia bound to the HNEC was evaluated by subtracting all unbound conidia collected in the three washings from the total number of conidia added to the cells. After removal of unbound conidia, the wells were incubated for the indicated times at 37°C in a 5% CO_2 _atmosphere.

### TEM and confocal studies

HNEC infected with *A. fumigatus *conidia were fixed for 2 h at 4°C in 0.045 M cacodylate buffer, pH 7.4, containing 2.5% glutaraldehyde, and post-fixed in buffered 1% osmium tetroxide for 90 min, stained in 2% uranyl acetate and dehydrated through graded solutions of ethanol. Cell culture membranes were removed from the inserts and embedded in Epon. Semi-thin sections (1 μm) were stained with toluidine blue and examined by light microscopy to evaluate the specimen prior to thin sectioning. Ultrathin sections (80 nm) were examined on a transmission electron microscope (Philips EM 301) at an acceleration of 80 kV and a final magnification ranging from × 2500 to × 30 000.

For the confocal microscopy study, freshly harvested conidia were labeled with fluorescein isothiocyanate (FITC) (Sigma, Saint-Quentin Fallavier, France) in 0.1 M carbonate buffer for 1 h with shaking at 37°C. The HNEC cells with FITC-labeled conidia were fixed for 1 h at 4°C in 2% paraformaldehyde, permeabilized with 2% methanol for 1 h at -20°C, and stored in PBS at 4°C. After incubation in 5% goat serum and 5% human serum in PBS to avoid non-specific binding of antibodies, HNEC were incubated for 90 min in 1:50 diluted mouse monoclonal anti-human cytokeratin specific antibody (mAb) (Immunotech, Marseille, France) to label HNEC. They were then washed and finally incubated for 90 min with 1:200 diluted rhodamine-conjugated goat anti-mouse antibody (Immunotech). After washing with PBS, the membranes with HNEC were removed from the inserts, mounted in Mowiol (Sigma), and examined with a Zeiss Laser Scanning 410 Microscope (LSM) (Göttingen, Germany). Images were obtained with LSM 3.98 Software (Zeiss) and further processed with Adobe Photoshop software.

### Immunolabeling with LAMP1 marker

To study the late endosome/lysosome compartment, HNEC were fixed for 1 h at 4°C in 2% paraformaldehyde and permeabilized with PBS containing 0.05% saponin as described previously [6]. HNEC were then incubated for 30 min at room temperature with a 1:100 diluted mouse anti-human LAMP1 mAb specific for human lysosomal membrane associated protein (BD Pharmingen, Franklin Lakes, NJ, USA). Purified mouse IgG1 isotype (Pharmingen) was used as a negative control. After washing, HNEC were incubated for 30 min with 1:200 diluted anti-mouse Texas-red-conjugated secondary antibody (Jackson ImmunoResearch Laboratories, West Grove, PA, USA). After washing, cells were mounted in Mowiol, sealed on microscope slides with nail varnish, and stored at 4°C until observation. Slides were examined with a fluorescence Leica DL microscope coupled to a Coolsnap Cf monochrome camera (Photometrics, Roper Scientific, Evry, France). Images were obtained with the Metaview program (Universal Imaging Corporation, Downingstown, PA, USA) and further processed with Adobe Photoshop software.

### Number of HNEC-associated conidia

HNEC cultures with unlabeled conidia were dissociated for 30 min at 37°C with 0.1% collagenase (Sigma, France) and 15 min at 37°C with 0.25% trypsin (Sigma, France). Reactions were stopped with 10% FCS. After centrifugation (5 min, 500 g), cells were resuspended, smeared and stained with May-Grünwald-Giemsa. One hundred randomly chosen HNEC were examined microscopically to calculate the number of HNEC with at least one associated conidium. We also determined the average number of conidia associated with each HNEC by counting 100 HNEC associated with at least one conidium. The experiments were repeated twice for each fungal species with two different primary cultures.

### Percentage of phagocytosed conidia and viability of conidia

To quantify phagocytosis among the bound *A. fumigatus *conidia, we used FITC-labeled conidia and an additional staining of non-phagocytosed conidia adapted from the method of Sturtevant *et al*. [29]. For specific labeling of non-phagocytosed conidia, HNEC were incubated for 30 min with 1:50 diluted rabbit anti-*A. fumigatus *conidia antiserum. After washing, HNEC were incubated for 30 min with 1:200 diluted Texas-red-conjugated anti-rabbit antibody (Sigma, France). Therefore, phagocytosed conidia, inaccessible to the anti-conidia antibody, were only FITC-labeled, whereas non-phagocytosed conidia were labeled with both FITC and Texas-red. Samples were viewed with a Zeiss Axiophot microscope equipped with epifluorescence filters. Extracellular and intracellular conidia were counted after merging the red and green channels in eclipse. The percentage of phagocytosed conidia was calculated as: number of FITC-positive and Texas-red-negative conidia/number of FITC-positive conidia × 100.

To quantify phagocytosed conidia of both fungal species we used the fluorescent brightener Blankophor-P-fluessig^® ^(4,4'-Bis [(4-anilino-6-substituted-1,3,5-trazine-2-yl)] amino stilbene-2,2'-disulfonic acid), kindly provided by Bayer, Leverkusen, Germany. This brightener is a stilbene compound like Calcofluor white and Uvitex, and presents no toxicity to mammalian cells [22]. The brightener stains only external conidia. Tenμl of the dye was diluted in 1 ml of culture medium and 300 μl of this dilution were added to the wells and incubated for 10 min at 37°C to allow staining of non-internalized conidia. The wells were washed twice to recover any remaining unattached conidia and to remove the dye. The HNEC were then lysed with 300 μl distilled water, and the remaining cells were scraped from the inserts and recovered in 300 μl. On excitation with ultraviolet light below 400 nm, the brightener emits a very intense bluish yellowish white light [31]. The filter combination included a barrier filter at 420 nm. Thus, the blue conidia were counted as non-internalized whereas the white conidia were assumed to have been internalized.

To test the viability of conidia obtained after brightener staining, propidium iodide (PI) staining (Sigma) was used. The same field was examined successively using different filter combinations. For the brightener staining, the filter combination included a barrier filter at 420 nm and allowed excitation at wavelengths lower than 400 nm. The other filter used was λ excitation at 540 nm and λ emission ≥ 570 nm, for PI staining. Several fields were read to count 200 conidia. Images were obtained using an Axioskop 40 microscope (Zeiss) with a digital camera DX20H (Kappa, Donneville, France) fitted to the microscope.

### Statistical analysis

Statistical analysis was performed using EPI-INFO 6.04c computer software (Centers for Disease Control and Prevention, Atlanta, GA, USA). Each value was derived from triplicate data points and was expressed as the mean ± SEM. The mean counts of *A. fumigatus *and *P. chrysogenum *conidia were compared using the Student's t test or Kruskall-Wallis test. A *p *value < 0.05 was considered to be statistically significant.

## Authors' contributions

FB, KG, OI-G, KK, CC, EE, and SB made substantial contributions to the conception and design of the study, and the acquisition, analysis and interpretation of the data. FB, KG, KK, VE, and AC were involved in cell culture growth, as well as development and biological tests. FB, OI-G, and SB were responsible for writing the manuscript and revising it critically for intellectual content, and gave final approval of the version to be submitted. FB, AC, CC, EE, and SB conceived the original study. All authors read and approved the final manuscript.
